# Eosinophils in lung cancer: from inflammatory cytokines to immunotherapeutic biomarkers

**DOI:** 10.3389/fimmu.2026.1797055

**Published:** 2026-04-28

**Authors:** Zhen Wang, Fuzhi Jiao, Jing Yuan, Shengnan Zhang, Fenglei Shi

**Affiliations:** 1 Department of Traditional Chinese Medicine, Qingdao Municipal Hospital, Qingdao, Shandong, China; 2 School of Biological and Chemical Engineering, Qingdao Technical College, Qingdao, Shandong, China

**Keywords:** biomarkers, eosinophils, immune checkpoint inhibitors, immunotherapy, inflammatory cytokine, lung cancer, metastasis, tumor microenvironment

## Abstract

Eosinophils, traditionally viewed as effector cells in allergic and parasitic responses, have emerged as multifaceted regulators within the tumor microenvironment (TME). In lung cancer, eosinophils demonstrate complex and context-dependent functions, shaped by chemokines, cytokines, and tumor-derived signals such as CCL11 and IL-33. Recent studies indicate that eosinophils may either promote anti-tumor immunity, by enhancing CD8^+^ T cell infiltration, secreting cytotoxic granules, and cooperating with IL-33, or facilitate tumor progression through recruitment of regulatory T cells, immune suppression, and expression of immunoregulatory enzymes like IDO. Moreover, eosinophil abundance in tumor tissues and peripheral blood has been associated with both favorable and unfavorable prognostic outcomes in lung cancer patients. Notably, elevated eosinophil counts correlate with improved responses to immune checkpoint inhibitors (ICIs), positioning them as potential biomarkers for immunotherapy efficacy. However, distinctions between tumor-infiltrating and circulating eosinophils, as well as their dualistic roles in metastasis and immune modulation, remain incompletely understood. This review summarizes current advances in understanding eosinophil biology in lung cancer and underscores their promise as diagnostic and therapeutic targets in precision immuno-oncology.

## 1 Introduction

Eosinophils are an important leukocyte population that helps maintain immune homeostasis. They are mainly distributed in the blood, lungs, mammary glands, gastrointestinal tract, and reproductive organs (1, 2). During allergic responses, helminth infection, bacterial or viral infection, or certain cancers, eosinophil counts typically increase markedly. Parasitic infection in mammals can elicit a robust Th2-like response, characterized by high expression of IL-4 and eosinophilia (3, 4). In addition, eosinophils participate in systemic metabolism, immunoregulation, neuronal regulation, and tissue remodeling and development (3, 5). In recent years, studies have shown that CCL11 and CCL24, secreted by tumor cells, macrophages, or fibroblasts, can promote eosinophil homing and recruitment into the tumor microenvironment, thereby influencing tumor initiation and progression (6, 7). Eosinophilia may exert either favorable or unfavorable effects on prognosis in cancer patients, yet this issue remains controversial; for example, in non-small cell lung cancer patients receiving immunotherapy, increased eosinophils are positively associated with improved survival (8). Moreover, eosinophils have been proposed as a potential biomarker for monitoring responses to tumor immunotherapy (9, 10). This review focuses on the roles of eosinophils in lung cancer and advances in mechanistic studies, discusses their functions in lung cancer immunity, and evaluates their potential value as clinical indicators of immunotherapy efficacy.

## 2 Characteristics of eosinophils

Eosinophils originate from multipotent and lineage-committed hematopoietic progenitor cells, and their development is regulated by various receptors, growth factors, and inhibitory cytokines (11, 12). Classic cytokines that promote eosinophil differentiation include IL-5, granulocyte-macrophage colony-stimulating factor (GM-CSF), and IL-3. These hematopoietic growth factors primarily activate T cells, mast cells, and bone marrow progenitor cells to synthesize and secrete them, which then act on multipotent hematopoietic progenitor cells, eosinophil progenitors, immature eosinophils, and mature eosinophils (11, 13). Correspondingly, these growth factors not only drive the proliferation of eosinophil progenitor cells but also regulate the migration, differentiation, survival, and activation of mature eosinophils (14). Eosinophils may reshape the tumor microenvironment and promote tumor progression through interactions with other immune cells or tumor cells (15, 16). Eosinophils can recruit and activate various stromal cells by releasing inflammatory mediators and stimulating local immune cells, thereby initiating fibrosis, angiogenesis, and tissue remodeling—for example, by interacting with fibroblasts or epithelial cells (17, 18). Eosinophils promote angiogenesis and fibrotic remodeling through direct interactions with endothelial cells and fibroblasts. Eosinophil-derived VEGF-A and MMP-9 can enhance endothelial sprouting by increasing vascular permeability and facilitating extracellular matrix (EMC) degradation (19, 20). Besides, TNF-α and IL-1β released by eosinophils may activate NF-κB signaling in stromal and endothelial cells, thereby sustaining a pro-angiogenic inflammatory microenvironment (15, 21–23). Eosinophil-derived IL-13 may drive fibroblast activation through STAT6 signaling, promoting myofibroblast differentiation and matrix production, whereas TGF-β1 can further amplify SMAD2/3-dependent EMC deposition and tissue stiffening (24–26). These findings suggest that eosinophils may active angiogenesis and fibrosis reprogramming in the lung tumor microenvironment.

Furthermore, eosinophils release pro-inflammatory cytokines that exacerbate tissue inflammation and contribute to tissue repair dysfunction (27). They also secrete a variety of cytokines, chemokines, growth factors, and other soluble mediators to modulate the tumor microenvironment and influence disease progression (15). For instance, eosinophils cooperate with IL-33 to suppress tumor cell proliferation in colorectal cancer via enhancement of anti-tumor immunity (28). Preclinical animal studies have shown that activated eosinophils secrete large quantities of chemokines such as CCL5, CXCL9, and CXCL10, which increase CD8^+^ T cell infiltration into tumor tissues and contribute to tumor suppression (15, 29). These cytotoxic mediators can directly affect tumor cell viability and modulate host anti-tumor immune responses, thereby influencing tumor immune surveillance and defense (6, 15). Moreover, reports have indicated that eosinophils can promote tumor immune escape by expressing the enzyme indoleamine 2,3-dioxygenase (IDO). In NSCLC patients, eosinophils expressing IDO were detected in peripheral blood. Their presence was associated with poor overall survival in NSCLC patients, suggesting that eosinophils may also play a role in facilitating tumor progression (30). Eosinophils are recruited into tumor tissues through inflammation induced by tumor cells, as well as by immune cells within or surrounding the tumor microenvironment, such as neutrophils, mast cells, and dendritic cells, which secrete chemotactic factors that guide eosinophil migration. Eosinophils are attracted by tumor-derived GM-CSF and CCL11, which promote their mobilization, activation, and directional transport toward the tumor tissue (15, 31). NSCLC models have shown that cytokines, such as IL-4, IL-5, IL-10, and IL-13, may effectively enhance eosinophil recruitment to tumor sites (32).

## 3 Eosinophilia in lung cancer progression

Patients with eosinophilic pleural effusion (EPE) had better prognosis than those without EPE, which has been identified as an independent risk factor for diagnosing malignant pleural effusion and assessing its prognosis, with notable clinical value (33). Moreover, tumor-associated tissue eosinophilia (TATE) and tumor-associated blood eosinophilia (TABE) have been observed across various anatomical sites and histological types of tumors, occurring either concurrently or independently. Elevated TATE often suggests a favorable prognosis, while TABE without concurrent TATE may indicate tumor dissemination and poor prognosis (34, 35). Recent research has employed TATE/TABE levels as potential indicators for tumor grading (36). Eosinophil peroxidase (EPO), a cytotoxic granule protein, is commonly used as a biomarker for eosinophils (37). In lung cancer, EPO expression was significantly higher in tumor tissues than in adjacent normal tissues (24). Overexpression of EPO correlated with advanced pathological stage and lymph node metastasis. Patients with low EPO expression exhibited prolonged survival, whereas those with high EPO levels had shorter survival durations. Furthermore, EPO has been identified as an independent prognostic factor in lung cancer. Elevated EPO expression may thus serve as a negative prognostic biomarker in lung cancer patients (38).

EPO may also participate directly in remodeling the inflammatory and redox landscape of the lung cancer microenvironment (24, 39). As a heme peroxidase released by activated eosinophils, EPO utilizes hydrogen peroxide and halides/pseudohalides to generate highly reactive oxidizing intermediates, thereby providing a plausible biochemical link between eosinophil infiltration and the elevated oxidative stress already recognized in lung cancer tissues (13, 40, 41). Although direct causal evidence in lung cancer remains limited, findings from airway epithelial models indicate that EPO can stimulate epithelial cells to upregulate inflammatory and remodeling-associated mediators, including GM-CSF, thymic stromal lymphopoietin (TSLP), TGF-β1 and ADAM33, and can promote epithelial activation through PI3K-dependent signaling (42, 43). In lung cancer, such EPO -driven oxidant stress may reasonably be expected to cooperate with pre-existing tumor-derived ROS to activate redox-sensitive transcriptional programs, particularly NF-κB, thereby amplifying expression of IL-6, TNF-α, CXCL8 and other pro-tumorigenic inflammatory mediators (44, 45). NF-κB-centered inflammatory circuits are known to sustain survival signaling, angiogenesis, invasion and therapy resistance, whereas IL-6/STAT3 signaling further promotes epithelial–mesenchymal transition, metastatic plasticity and persistent inflammatory reinforcement in lung cancer cells (46–49). Moreover, CXCL8 and IL-8 induction could reshape the granulocytic and myeloid composition of the tumor microenvironment, thereby intensifying paracrine inflammatory crosstalk rather than merely reflecting eosinophil abundance (50–52). Thus, EPO may also function as an active amplifier of oxidative and cytokine-driven signaling within the tumor microenvironment. This perspective may help explain why high EPO expression is associated with adverse clinicopathological features in some lung cancer cohorts, despite the antitumor activities reported for eosinophils in other settings. Nevertheless, this proposed EPO–ROS–NF-κB/IL-6/STAT3 axis requires direct validation in lung cancer-specific coculture, spatial, and *in vivo* models (53–55).

## 4 Eosinophils in lung cancer cell metastasis

Cancer cell metastasis is the primary cause of cancer-related mortality (56, 57). However, the biological mechanisms underlying lung cancer metastasis remain incompletely understood. A growing body of evidence indicates that tumor cell–derived chemokines CCL24 and CCL11, as well as CCL11 produced by macrophages, fibroblasts, and even eosinophils themselves, promote eosinophil recruitment to the tumor microenvironment through binding to CCR3 expressed on the eosinophil surface (6, 58). In addition, tumor-derived CCL3 and CCL5 can further support eosinophil migration toward tumor sites (15, 59). Researchers employed multiple lung metastasis models and demonstrated that activation of pulmonary group 2 innate lymphoid cells (ILC2s) suppresses NK cell–mediated innate antitumor immunity, thereby promoting lung cancer metastasis and increasing mortality (60, 61). IL-33 activates ILC2s, which in turn drive type 2 innate inflammation, suppress interferon-γ production, and impair NK cell cytotoxic function. Importantly, ILC2-mediated suppression of NK cell activity is dependent on IL-5–induced pulmonary eosinophilia, which ultimately constrains NK cell metabolic capacity. Thus, eosinophils serve as a critical cellular bridge in the IL-33/ILC2 axis that facilitates lung cancer metastasis (62).

Notably, IL-33 exerts dual effects on tumor development. IL-33 can stimulate macrophages to produce TNF-α, thereby upregulating the expression of the IL-33–specific receptor suppression of tumorigenicity 2 (ST2) on NK cells, which plays a critical role in NK cell activation (63, 64). IL-33 therapy may induce eosinophils and CD8^+^ T cells to secrete CCL5, thereby promoting NK cell recruitment to the tumor microenvironment (63). Systemic activation and local accumulation of NK cells can trigger a robust anti-tumor rejection response in the lung. In addition, IL-33 administration has been shown to effectively suppress lung metastasis of murine breast cancer (63). Similarly, in subcutaneous melanoma models, intratumoral injection of IL-33 significantly delayed tumor growth. Intranasal administration of IL-33 induced ST2-dependent eosinophil recruitment to the lung, thereby effectively preventing pulmonary metastasis following intravenous injection of melanoma cells (65, 66). Consistently, ST2-deficient mice exhibited a significantly higher burden of lung metastases compared with wild-type mice, which was associated with reduced numbers of pulmonary eosinophils. These studies demonstrated that IL-33 can directly activate eosinophils, enabling them to efficiently kill target melanoma cells. These findings indicate that eosinophils possess direct anti-tumor cytotoxic activity following IL-33–based therapy (67).

IL-5 is widely recognized as a key cytokine responsible for maintaining eosinophil proliferation and survival (68, 69). In murine models of ectopic lung metastasis and intravenous tumor cell injection, IL-5 promotes metastatic colonization by recruiting pre-existing eosinophils and modulating inflammatory and immune cell populations within the distal pulmonary microenvironment (16, 70). Genetic deficiency of IL-5 confers significant protection against pulmonary metastasis of multiple tumor types, including lung cancer, colorectal cancer, and melanoma (15, 71). Neutralization of IL-5 similarly protects recipient mice from tumor cell metastasis, whereas reconstitution of IL-5 or adoptive transfer of eosinophils into IL-5–deficient mice restore metastatic potential (15, 24). Notably, IL-5 deficiency does not affect the growth or intrinsic metastatic capacity of primary tumors (15). Eosinophils produce CCL22, which facilitates the recruitment of Tregs to the lung (72). During the early stages of metastasis, Tregs suppress IFN-γ production by NK cells and inhibit M1-polarized macrophages, thereby establishing a pro-tumorigenic microenvironment conducive to metastatic seeding (73). Consistently, IL-5 functions as a central regulator of eosinophil expansion, and genetic deletion or neutralization of IL-5 markedly attenuates eosinophil recruitment to the lung, resulting in enhanced tumor metastasis (74). Therefore, monitoring the functional role of pulmonary eosinophils in lung cancer may provide novel insights for the development of innovative cancer immunotherapeutic strategies. Some studies have proposed that eosinophil recruitment to pulmonary metastatic sites represents a defining feature of breast cancer lung metastasis. This process is regulated by G protein–coupled receptor (GPCR) signaling, yet occurs independently of CCR3 signaling (75).

## 5 Eosinophils as potential biomarkers for monitoring the efficacy of lung cancer immunotherapy

Immunotherapy has become a cornerstone in the treatment of advanced lung cancer and occupies a central role in first-line therapeutic regimens (76). Lung cancer immunotherapy primarily refers to the use of immune checkpoint inhibitors (ICIs), particularly inhibitors targeting programmed death-ligand 1 (PD-L1) and its receptor programmed cell death protein 1 (PD-1) (77). PD-L1 is predominantly expressed in proximity to IFN-γ–producing T cells and exhibits marked spatial and temporal heterogeneity within tumor tissues (78). In addition, clinical responses to immunotherapy vary substantially among patients, and immune-related adverse events remain a significant concern. For example, patients with NSCLC complicated by interstitial lung disease are at increased risk of developing immune-related pneumonitis following immunotherapy (79). At present, clinically reliable biomarkers capable of accurately predicting immunotherapy efficacy are still lacking. Although the relationship between eosinophils and cancer immunotherapy remains incompletely understood, both eosinophil infiltration within tumor tissues and elevated eosinophil counts in the peripheral blood of cancer patients have been clinically documented (30). Eosinophil granule proteins are cationic proteins and are regarded as key effector molecules mediating the antitumor activity of eosinophils. In addition, eosinophils secrete a broad range of cytokines, chemokines, and other soluble mediators, including IL-4, IL-6, IFN-γ, and TNF, which play critical roles in tumor initiation and progression (6, 80).

Eosinophils may also represent actionable immune intermediates in rational combination strategies designed to enhance checkpoint blockade. Eosinophils acquire a more immunostimulatory phenotype capable of promoting type 1 T-cell immunity via GM-CSF–IRF5 signaling axis (81). GM-CSF signaling activates IRF5 in eosinophils and induces the expression of multiple immune mediators, including CCL2, CCL24, IL-1α, IL-1β, IL-13, and TNF-α, thereby strengthening intratumoral T-cell-oriented inflammatory programs (82, 83). Importantly, this eosinophil-activating circuit is antagonized by IL-10, suggesting that the balance between GM-CSF-driven activation and IL-10-mediated restraint may critically determine whether eosinophils support productive anti-tumor immunity during ICI therapy (84, 85). These findings raise the possibility that eosinophil dynamics may reflect not only host immune status, but also the degree of cytokine-driven immune reprogramming achieved by therapy. Another potential direction for combination treatment involves epithelial alarmin–dependent eosinophil priming beyond the IL-33/IL-5 axis (61). Thymic stromal lymphopoietin (TSLP) has been shown to prolong eosinophil survival, enhance the expression of adhesion molecules ICAM-1 and CD18, and induce the release of inflammatory mediators such as IL-6, CXCL1, CXCL8, and CCL2 through p38 MAPK, ERK, and NF-κB signaling (86, 87).

**Figure 1:**
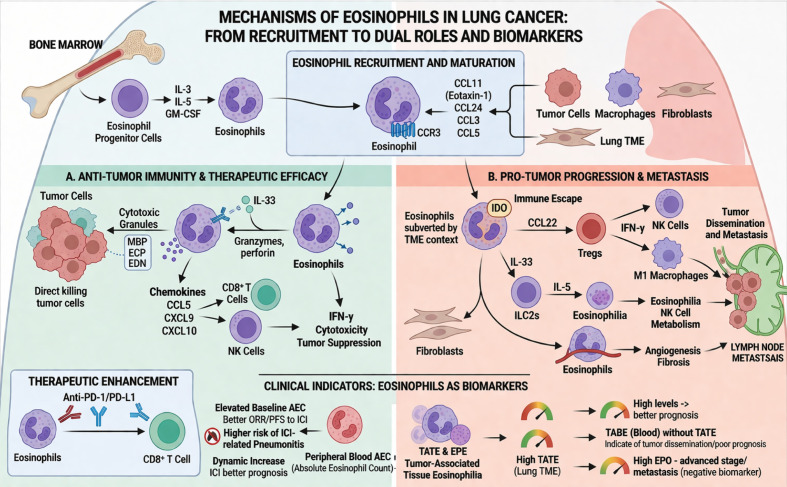
Role of eosinophils in lung cancer progression, metastasis, and immune checkpoint inhibition.

Among leukocyte subsets, only relative eosinophil counts were associated with clinical responses to PD-1/PD-L1 blockade (88). In NSCLC patients receiving ICIs, particularly those treated with ICI monotherapy, eosinophil counts during treatment was associated with delayed treatment failure (89), 27% of lung cancer patients developed peripheral blood eosinophilia during immunotherapy. Patients with marked eosinophilia exhibited superior overall response rates, higher overall survival, and prolonged progression-free survival. Moreover, increase in peripheral blood eosinophil proportion within two weeks of treatment initiation was positively correlated with objective responses to PD-1 blockade and improved prognosis. Hence, peripheral blood eosinophilia may serve as a predictive biomarker for immunotherapy outcomes in lung cancer patients (90). Patients received immunotherapy reported the developed immune checkpoint inhibitor–related pneumonitis. Baseline peripheral blood absolute eosinophil counts (AECs) were significantly higher in patients who developed ICI-related pneumonitis. In addition, patients with elevated AECs exhibited higher objective response rates and longer median progression-free survival (91). In murine lung cancer metastasis models, IL-33 enhanced antitumor immune responses characterized by augmented NF-κB signaling, accompanied by increased activation, proliferation, and infiltration of CD8^+^ T cells, NK cells, and eosinophils (67, 92). Moreover, IL-33 was shown to slow the metastatic spread of tumor cells to distant organs. However, the conditions under which IL-33 therapy enhances eosinophil-mediated antitumor activity require further investigation (93). Radiotherapy promotes eosinophil infiltration within the lung tumor microenvironment (94). Eosinophils subsequently enhance CD8^+^ T cell–mediated antitumor immunity, thereby improving the therapeutic efficacy of ICIs. Similarly, abundant eosin (92)ophil infiltration in metastatic triple-negative breast cancer with favorable responses to immunotherapy (95). Eosinophil accumulation in this context was dependent on CD4^+^ T cells and eosinophil-stimulating cytokines IL-5 and IL-33, and eosinophils enhanced immunotherapeutic responses by promoting CD8^+^ T cell activation (**Figure 1**).

## 6 Conclusion

Eosinophils have emerged as pivotal, yet paradoxical, regulators within the lung cancer tumor microenvironment. Far from being mere effectors of allergic responses, they exhibit profound functional plasticity driven by cytokines such as IL-33, IL-5, and CCL11. While eosinophils can facilitate tumor progression and metastasis by recruiting regulatory T cells, promoting angiogenesis via EPO-mediated oxidative stress, and suppressing NK cell activity, they simultaneously possess potent anti-tumor capabilities. Specifically, activated eosinophils enhance CD8^+^ T cell infiltration and exert direct cytotoxicity, highlighting a delicate balance between pro- and anti-tumorigenic roles. This duality is heavily context-dependent, influenced by distinct chemokine gradients and spatial localization within tumor tissues versus peripheral circulation.

Clinically, eosinophil dynamics offer significant promise as accessible biomarkers for precision immuno-oncology. Elevated peripheral and tumor-infiltrating eosinophil counts consistently correlate with improved responses to immune checkpoint inhibitors and prolonged survival in non-small cell lung cancer patients. However, discrepancies between tissue and blood eosinophilia, alongside their complex involvement in immune-related adverse events like pneumonitis, necessitate further mechanistic clarification. Future research must dissect the specific molecular switches governing eosinophil polarization. Ultimately, harnessing eosinophil biology through targeted combination therapies could optimize immunotherapeutic efficacy, transforming these multifaceted cells into valuable tools for diagnostic stratification and therapeutic intervention in lung cancer management.

## References

[1] Lombardi C Berti A Cottini M . The emerging roles of eosinophils: implications for the targeted treatment of eosinophilic-associated inflammatory conditions. Curr Res Immunol. (2022) 3:42–53. doi: 10.1016/j.crimmu.2022.03.002. PMID: 35496822 PMC9040157

[2] Gurtner A Crepaz D Arnold IC . Emerging functions of tissue-resident eosinophils. J Exp Med. (2023) 220:e20221435. doi: 10.1084/jem.20221435. PMID: 37326974 PMC10276195

[3] Wechsler ME Munitz A Ackerman SJ Drake MG Jackson DJ Wardlaw AJ . Eosinophils in health and disease: a state-of-the-art review. Mayo Clin Proc. (2021) 96:2694–707. doi: 10.1016/j.mayocp.2021.04.025. PMID: 34538424

[4] Guth C Schumacher PP Vijayakumar A Borgmann H Balles H Koschel M . Eosinophils are an endogenous source of interleukin-4 during filarial infections and contribute to the development of an optimal T helper 2 response. J Innate Immun. (2024) 16:159–72. doi: 10.1159/000536357. PMID: 38354709 PMC10932553

[5] Diny NL Yamada Y Zimmermann N . Editorial: update on eosinophil-associated diseases. (2025) 6:1740057. doi: 10.3389/falgy.2025.1740057, PMID: 41415873 PMC12708251

[6] Lopez-Perez D Prados-Lopez B Galvez J Leon J Carazo A . Eosinophils in colorectal cancer: emerging insights into anti-tumoral mechanisms and clinical implications. (2024) 25:6098. doi: 10.3390/ijms25116098, PMID: 38892286 PMC11172675

[7] Lim SJ . CCL24 signaling in the tumor microenvironment. Adv Exp Med Biol. (2021) 1302:91–8. doi: 10.1007/978-3-030-62658-7_7. PMID: 34286443

[8] Tanizaki J Haratani K Hayashi H Chiba Y Nakamura Y Yonesaka K . Peripheral blood biomarkers associated with clinical outcome in non-small cell lung cancer patients treated with nivolumab. J Thorac Oncol. (2018) 13:97–105. doi: 10.1016/j.jtho.2017.10.030. PMID: 29170120

[9] Suzuki R Ohkuma R Watanabe M Mura E Tsurui T Iriguchi N . Eosinophils and the efficacy of immune checkpoint inhibitors across multiple cancers: a retrospective study. Biomedicines. (2025) 13:3029. doi: 10.3390/biomedicines13123029. PMID: 41463041 PMC12730934

[10] Qin Y Yao S Huang H Xiao Y Wang J She L . Peripheral eosinophils and immunotherapy response in patients with recurrent or metastatic HNSCC. Sci Rep. (2025) 15:17351. doi: 10.1038/s41598-025-01457-6. PMID: 40389501 PMC12089525

[11] Tao Z Zhu H Zhang J Huang Z Xiang Z Hong T . Recent advances of eosinophils and its correlated diseases. Front Public Health. (2022) 10:954721. doi: 10.3389/fpubh.2022.954721. PMID: 35958837 PMC9357997

[12] Valent P Degenfeld-Schonburg L Sadovnik I Horny H-P Arock M Simon H-U . Eosinophils and eosinophil-associated disorders: immunological, clinical, and molecular complexity. Semin Immunopathol. (2021) 43:423–38. doi: 10.1007/s00281-021-00863-y. PMID: 34052871 PMC8164832

[13] Sanchez Santos A Socorro Avila I Galvan Fernandez H Cazorla Rivero S Lemes Castellano A Cabrera Lopez C . Eosinophils: old cells, new directions. Front Med (Lausanne). (2024) 11:1470381. doi: 10.3389/fmed.2024.1470381. PMID: 39886455 PMC11780905

[14] Antosz K Batko J Błażejewska M Gawor A Sleziak J Gomułka K . Insight into IL-5 as a potential target for the treatment of allergic diseases. (2024) 12:1531. doi: 10.3390/biomedicines12071531, PMID: 39062104 PMC11275030

[15] Ghaffari S Rezaei N . Eosinophils in the tumor microenvironment: implications for cancer immunotherapy. J Transl Med. (2023) 21:551. doi: 10.1186/s12967-023-04418-7. PMID: 37587450 PMC10433623

[16] Grisaru-Tal S Rothenberg ME Munitz A . Eosinophil-lymphocyte interactions in the tumor microenvironment and cancer immunotherapy. Nat Immunol. (2022) 23:1309–16. doi: 10.1038/s41590-022-01291-2. PMID: 36002647 PMC9554620

[17] Dunn JLM Caldwell JM Ballaban A Ben-Baruch Morgenstern N Rochman M Rothenberg ME . Bidirectional crosstalk between eosinophils and esophageal epithelial cells regulates inflammatory and remodeling processes. Mucosal Immunol. (2021) 14:1133–43. doi: 10.1038/s41385-021-00400-y. PMID: 33972688 PMC8380647

[18] Yang H-W Park J-H Jo M-S Shin J-M Kim DW Park I-H . Eosinophil-derived osteopontin induces the expression of pro-inflammatory mediators and stimulates extracellular matrix production in nasal fibroblasts: the role of osteopontin in eosinophilic chronic rhinosinusitis. (2022) 13:777928. doi: 10.3389/fimmu.2022.777928, PMID: 35309360 PMC8924074

[19] Ngaha TYS Zhilenkova AV Essogmo FE Uchendu IK Abah MO Fossa LT . Angiogenesis in lung cancer: understanding the roles of growth factors. Cancers (Basel). (2023) 15:4648. doi: 10.20944/preprints202308.1800.v2. PMID: 37760616 PMC10526378

[20] Tota M Łacwik J Laska J Sędek Ł Gomułka K . The role of eosinophil-derived neurotoxin and vascular endothelial growth factor in the pathogenesis of eosinophilic asthma. Cells. (2023) 12:1326. doi: 10.3390/cells12091326. PMID: 37174726 PMC10177218

[21] Wu S Hu Y Sui B . Promotion mechanisms of stromal cell-mediated lung cancer development within tumor microenvironment. Cancer Manag Res. (2025) 17:249–66. doi: 10.2147/cmar.s505549. PMID: 39957904 PMC11829646

[22] Cao Y Yi Y Han C Shi B . NF-κB signaling pathway in tumor microenvironment. Front Immunol. (2024) 15:1476030. doi: 10.3389/fimmu.2024.1476030. PMID: 39493763 PMC11530992

[23] Leone P Malerba E Susca N Favoino E Perosa F Brunori G . Endothelial cells in tumor microenvironment: insights and perspectives. Front Immunol. (2024) 15:1367875. doi: 10.3389/fimmu.2024.1367875. PMID: 38426109 PMC10902062

[24] Omero F Speranza D Murdaca G Cavaleri M Marafioti M Cianci V . The role of eosinophils, eosinophil-related cytokines and AI in predicting immunotherapy efficacy in NSCLC. (2025) 15:491. doi: 10.3390/biom15040491, PMID: 40305195 PMC12024677

[25] Chao H Zheng L Hsu P He J Wu R Xu S . IL-13RA2 downregulation in fibroblasts promotes keloid fibrosis via JAK/STAT6 activation. JCI Insight. (2023) 8:e157091. doi: 10.1172/jci.insight.157091. PMID: 36757802 PMC10070111

[26] Chen F Lyu L Xing C Chen Y Hu S Wang M . The pivotal role of TGF-β/Smad pathway in fibrosis pathogenesis and treatment. Front Oncol. (2025) 15:1649179. doi: 10.3389/fonc.2025.1649179. PMID: 40969268 PMC12440922

[27] Day KS Rempel L Rossi FMV Theret M . Origins and functions of eosinophils in two non-mucosal tissues. (2024) 15:1368142. doi: 10.3389/fimmu.2024.1368142, PMID: 38585275 PMC10995313

[28] Kienzl M Hasenoehrl C Valadez-Cosmes P Maitz K Sarsembayeva A Sturm E . IL-33 reduces tumor growth in models of colorectal cancer with the help of eosinophils. Oncoimmunology. (2020) 9:1776059. doi: 10.1080/2162402x.2020.1776059. PMID: 32923137 PMC7458617

[29] Abdul-Rahman T Ghosh S Badar SM Nazir A Bamigbade GB Aji N . The paradoxical role of cytokines and chemokines at the tumor microenvironment: a comprehensive review. Eur J Med Res. (2024) 29:124. doi: 10.1186/s40001-024-01711-z. PMID: 38360737 PMC10868116

[30] Grisaru-Tal S Itan M Klion AD Munitz A . A new dawn for eosinophils in the tumour microenvironment. Nat Rev Cancer. (2020) 20:594–607. doi: 10.1038/s41568-020-0283-9. PMID: 32678342

[31] Bhattacharyya S Oon C Diaz L Sandborg H Stempinski ES Saoi M . Autotaxin-lysolipid signaling suppresses a CCL11-eosinophil axis to promote pancreatic cancer progression. Nat Cancer. (2024) 5:283–98. doi: 10.1038/s43018-023-00703-y. PMID: 38195933 PMC10899115

[32] Sibille A Corhay JL Louis R Ninane V Jerusalem G Duysinx B . Eosinophils and lung cancer: from bench to bedside. Int J Mol Sci. (2022) 23:5066. doi: 10.3390/ijms23095066. PMID: 35563461 PMC9101877

[33] Takeuchi E Okano Y Machida H Atagi K Kondou Y Kadota N . Eosinophilic pleural effusion due to lung cancer has a better prognosis than non-eosinophilic Malignant pleural effusion. Cancer Immunol Immunother. (2022) 71:365–72. doi: 10.1007/s00262-021-02994-5. PMID: 34170380 PMC8783892

[34] Caruso R Caruso V Rigoli L . Eosinophil ETosis and cancer: ultrastructural evidence and oncological implications. (2025) 17:3250. doi: 10.20944/preprints202508.0641.v1 PMC12523443 41097776

[35] Choudhary N Sarode GS Yuwanati M Maniyar N Sarode SC Gadbail AR . Tumor associated tissue eosinophilia in oral squamous cell carcinoma: a systematic review and meta-analysis. J Oral Biol Craniofac Res. (2021) 11:33–9. doi: 10.1016/j.jobcr.2020.11.012. PMID: 33344159 PMC7736986

[36] Verma F Juneja S Tandon A Shetty DC . Tumor-associated tissue eosinophilia versus tumor associated blood eosinophilia: a ratio of diagnostic importance in oral squamous cell carcinoma. J Cancer Res Ther. (2020) 16:581–6. doi: 10.4103/jcrt.jcrt_848_18. PMID: 32719271

[37] Tang M Charbit AR Johansson MW Jarjour NN Denlinger LC Raymond WW . Utility of eosinophil peroxidase as a biomarker of eosinophilic inflammation in asthma. J Allergy Clin Immunol. (2024) 154:580–591.e586. doi: 10.1016/j.jaci.2024.03.023. PMID: 38663815 PMC12765372

[38] Ye L Wang H Li H Liu H Lv T Song Y . Eosinophil peroxidase over-expression predicts the clinical outcome of patients with primary lung adenocarcinoma. J Cancer. (2019) 10:1032–8. doi: 10.7150/jca.24314. PMID: 30854109 PMC6400814

[39] Callander JK Charbit AR Khanna K Fahy JV Tang M Liegeois M . In office sampling of eosinophil peroxidase to diagnose eosinophilic chronic rhinosinusitis. Int Forum Allergy Rhinol. (2025) 15:36–44. doi: 10.1002/alr.23448. PMID: 39269218 PMC11697230

[40] Singh E Gupta A Singh P Jain M Muthukumaran J Singh RP . Exploring mammalian heme peroxidases: a comprehensive review on the structure and function of myeloperoxidase, lactoperoxidase, eosinophil peroxidase, thyroid peroxidase and peroxidasin. Arch Biochem Biophys. (2024) 761:110155. doi: 10.1016/j.abb.2024.110155. PMID: 39278306

[41] Lee E Hong JH . Oxidative stress defense module in lung cancers: molecular pathways and therapeutic approaches. Antioxid (Basel). (2025) 14:857. doi: 10.3390/antiox14070857. PMID: 40722961 PMC12291997

[42] Siddiqui S Bachert C Bjermer L Buchheit KM Castro M Qin Y . Eosinophils and tissue remodeling: relevance to airway disease. J Allergy Clin Immunol. (2023) 152:841–57. doi: 10.1016/j.jaci.2023.06.005. PMID: 37343842

[43] Xu L Huang X Chen Z Yang M Deng J . Eosinophil peroxidase promotes bronchial epithelial cells to secrete asthma-related factors and induces the early stage of airway remodeling. Clin Immunol. (2024) 263:110228. doi: 10.1016/j.clim.2024.110228. PMID: 38663494

[44] Zhao B Mei Y Yang J Ji P . Erythropoietin-regulated oxidative stress negatively affects enucleation during terminal erythropoiesis. Exp Hematol. (2016) 44:975–81. doi: 10.1016/j.exphem.2016.06.249. PMID: 27364565 PMC5035599

[45] Azad N Rojanasakul Y Vallyathan V . Inflammation and lung cancer: roles of reactive oxygen/nitrogen species. J Toxicol Environ Health B Crit Rev. (2008) 11:1–15. doi: 10.1080/10937400701436460. PMID: 18176884

[46] Zhang L Ludden CM Cullen AJ Tew KD Branco de Barros AL Townsend DM . Nuclear factor kappa B expression in non-small cell lung cancer. BioMed Pharmacother. (2023) 167:115459. doi: 10.1016/j.biopha.2023.115459. PMID: 37716117 PMC10591792

[47] Rajasegaran T How CW Saud A Ali A Lim JCW . Targeting inflammation in non-small cell lung cancer through drug repurposing. Pharm (Basel). (2023) 16:451. doi: 10.3390/ph16030451. PMID: 36986550 PMC10051080

[48] Liu J Liu Q Qian W Zong C Wang R . IL-6 promotes metastasis and EMT of non-small cell lung cancer cells by up-regulating FGL1 via STAT3 pathway. Transl Cancer Res. (2025) 14:3973–90. doi: 10.21037/tcr-2025-119. PMID: 40792131 PMC12335700

[49] Wang S Di Trapani G Tonissen KF . Expanding the armory for treating lymphoma: Targeting redox cellular status through thioredoxin reductase inhibition. Pharmacol Res. (2022) 177:106134. doi: 10.1016/j.phrs.2022.106134. PMID: 35189357

[50] Jung H Paust S . Chemokines in the tumor microenvironment: implications for lung cancer and immunotherapy. Front Immunol. (2024) 15:1443366. doi: 10.3389/fimmu.2024.1443366. PMID: 39114657 PMC11304008

[51] Shi J Li J Wang H Li X Wang Q Zhao C . Single-cell profiling of tumor-associated neutrophils in advanced non-small cell lung cancer. Lung Cancer (Auckl). (2023) 14:85–99. doi: 10.1016/j.iotech.2022.100320. PMID: 38025400 PMC10676108

[52] Zheng Y Cai J Ji Q Liu L Liao K Dong L . Tumor-activated neutrophils promote lung cancer progression through the IL-8/PD-L1 pathway. Curr Cancer Drug Targets. (2025) 25:294–305. doi: 10.2174/0115680096337237240909101904. PMID: 39354766 PMC11851149

[53] Chiu DK Zhang X Cheng BY Liu Q Hayashi K Yu B . Tumor-derived erythropoietin acts as an immunosuppressive switch in cancer immunity. Science. (2025) 388:eadr3026. doi: 10.1126/science.adr3026. PMID: 40273234 PMC12110762

[54] Ferrero JM Mograbi B Bouriga R Gal J Milano G . Rethinking EPO: A paradigm shift in oncology? Cancers (Basel). (2025) 17:3875. doi: 10.3390/cancers17233875. PMID: 41375075 PMC12691455

[55] Zhang Y Zhu Y Wang S Feng YC Li H . Erythropoietin receptor is a risk factor for prognosis: A potential biomarker in lung adenocarcinoma. Pathol Res Pract. (2023) 251:154891. doi: 10.21203/rs.3.rs-1211017/v3. PMID: 37844485

[56] Mello RM Gomez Ceballos D Sandate CR Wang S Jouffe C Agudelo D . BMAL1 and ARNT enable circadian HIF2α responses in clear cell renal cell carcinoma. Nat Commun. (2025) 16:5834. doi: 10.1038/s41467-025-60904-0. PMID: 40595592 PMC12215836

[57] Wang S Lamia KA . Integration of circadian and hypoxia signaling via non-canonical heterodimerization. FEBS Lett. (2026) 600:862–3. doi: 10.1002/1873-3468.70243. PMID: 41362097 PMC13022751

[58] Reichman H Karo-Atar D Munitz A . Emerging roles for eosinophils in the tumor microenvironment. Trends Cancer. (2016) 2:664–75. doi: 10.1016/j.trecan.2016.10.002. PMID: 28741505

[59] Wang J Man K Ng K-P . Emerging roles of C-C motif ligand 11 (CCL11) in cancers and liver diseases: Mechanisms and therapeutic implications. (2025) 26:4662. doi: 10.3390/ijms26104662, PMID: 40429807 PMC12111778

[60] Chen Y Ji X Qiu J Qiu J . The context-dependent role of group 2 innate lymphoid cells in lung diseases. Acta Biochim Biophys Sin (Shanghai). (2026) 58:120–36. doi: 10.3724/abbs.2025243. PMID: 41445394 PMC12862609

[61] Bahhar I Eş Z Köse O Turna A Günlüoğlu MZ Çakır A . The IL-25/ILC2 axis promotes lung cancer with a concomitant accumulation of immune-suppressive cells in tumors in humans and mice. Front Immunol. (2023) 14:1244437. doi: 10.3389/fimmu.2023.1244437. PMID: 37781372 PMC10540623

[62] Schuijs MJ Png S Richard AC Tsyben A Hamm G Stockis J . ILC2-driven innate immune checkpoint mechanism antagonizes NK cell antimetastatic function in the lung. Nat Immunol. (2020) 21:998–1009. doi: 10.1038/s41590-020-0745-y. PMID: 32747815 PMC7116357

[63] Qi L Zhang Q Miao Y Kang W Tian Z Xu D . Interleukin-33 activates and recruits natural killer cells to inhibit pulmonary metastatic cancer development. Int J Cancer. (2020) 146:1421–34. doi: 10.1002/ijc.32779. PMID: 31709531

[64] Choi M-R Sosman JA Zhang B . The Janus face of IL-33 signaling in tumor development and immune escape. (2021) 13:3281. doi: 10.3390/cancers13133281, PMID: 34209038 PMC8268428

[65] Feng Z Kuang Y Qi Y Wang X Xu P Chen X . Exogenous IL-33 promotes tumor immunity via macroscopic regulation of ILC2s. Sci Rep. (2024) 14:26140. doi: 10.1038/s41598-024-77751-6. PMID: 39478174 PMC11525627

[66] Turlej E Domaradzka A Radzka J Drulis-Fajdasz D Kulbacka J Gizak A . Cross-talk between cancer and its cellular environment-a role in cancer progression. Cells. (2025) 14:403. doi: 10.3390/cells14060403. PMID: 40136652 PMC11940884

[67] Lucarini V Ziccheddu G Macchia I La Sorsa V Peschiaroli F Buccione C . IL-33 restricts tumor growth and inhibits pulmonary metastasis in melanoma-bearing mice through eosinophils. Oncoimmunology. (2017) 6:e1317420. doi: 10.1080/2162402x.2017.1317420. PMID: 28680750 PMC5486175

[68] Nagase H Ueki S Fujieda S . The roles of IL-5 and anti-IL-5 treatment in eosinophilic diseases: Asthma, eosinophilic granulomatosis with polyangiitis, and eosinophilic chronic rhinosinusitis. Allergol Int. (2020) 69:178–86. doi: 10.1016/j.alit.2020.02.002. PMID: 32139163

[69] Buchheit KM Shaw D Chupp G Lehtimaki L Heffler E Finney-Hayward T . Interleukin-5 as a pleiotropic cytokine orchestrating airway type 2 inflammation: Effects on and beyond eosinophils. Allergy. (2024) 79:2662–79. doi: 10.1111/all.16303. PMID: 39359069

[70] Grisaru-Tal S Jacobsen EA Munitz A . Evolving role for eosinophils in cancer: From bench to bedside. Trends Cancer. (2025) 11:862–76. doi: 10.1016/j.trecan.2025.05.005. PMID: 40514310

[71] Deng S Clowers MJ Velasco WV Ramos-Castaneda M Moghaddam SJ . Understanding the complexity of the tumor microenvironment in K-ras mutant lung cancer: Finding an alternative path to prevention and treatment. (2020) 9:2019. doi: 10.3389/fonc.2019.01556, PMID: 32039025 PMC6987304

[72] Artham S Chang CY McDonnell DP . Eosinophilia in cancer and its regulation by sex hormones. Trends Endocrinol Metab. (2023) 34:5–20. doi: 10.1016/j.tem.2022.11.002. PMID: 36443206 PMC10122120

[73] Zaynagetdinov R Sherrill TP Gleaves LA McLoed AG Saxon JA Habermann AC . Interleukin-5 facilitates lung metastasis by modulating the immune microenvironment. Cancer Res. (2015) 75:1624–34. doi: 10.1158/0008-5472.can-14-2379. PMID: 25691457 PMC4401663

[74] Ikutani M Yanagibashi T Ogasawara M Tsuneyama K Yamamoto S Hattori Y . Identification of innate IL-5-producing cells and their role in lung eosinophil regulation and antitumor immunity. J Immunol. (2012) 188:703–13. doi: 10.4049/jimmunol.1101270. PMID: 22174445

[75] Grisaru-Tal S Dulberg S Beck L Zhang C Itan M Hediyeh-Zadeh S . Metastasis-entrained eosinophils enhance lymphocyte-mediated antitumor immunity. Cancer Res. (2021) 81:5555–71. doi: 10.1158/0008-5472.can-21-0839. PMID: 34429328

[76] Xiong A Wang J Zhou C . Immunotherapy in the first-line treatment of NSCLC: Current status and future directions in China. (2021) 11:2021. doi: 10.3389/fonc.2021.757993, PMID: 34900707 PMC8654727

[77] Dantoing E Piton N Salaün M Thiberville L Guisier F . Anti-PD1/PD-L1 immunotherapy for non-small cell lung cancer with actionable oncogenic driver mutations. Int J Mol Sci. (2021) 22:6288. doi: 10.3390/ijms22126288. PMID: 34208111 PMC8230861

[78] Okauchi S Shiozawa T Miyazaki K Nishino K Sasatani Y Ohara G . Association between peripheral eosinophils and clinical outcomes in patients with non-small cell lung cancer treated with immune checkpoint inhibitors. Pol Arch Intern Med. (2021) 131:152–60. doi: 10.20452/pamw.15776. PMID: 33491942

[79] Chen X Li Z Wang X Zhou J Wei Q Jiang R . Association of pre-existing lung interstitial changes with immune-related pneumonitis in patients with non-small lung cancer receiving immunotherapy. Support Care Cancer. (2022) 30:6515–24. doi: 10.1007/s00520-022-07005-6. PMID: 35411466

[80] Ramos-Casals M Flores-Chavez A Brito-Zerón P . Emerging role of eosinophils in immune-related adverse events related to therapy with immune checkpoint inhibitors. Pol Arch Intern Med. (2021) 131:16092. doi: 10.20452/pamw.16092. PMID: 34704705

[81] Gupta K Falta NA Spencer LA . Innate immune pairing: Eosinophils as hidden architects of T cell immunity. Cells. (2025) 14:1826. doi: 10.3390/cells14221826. PMID: 41294879 PMC12651172

[82] Roberts BK Collado G Barnes BJ . Role of interferon regulatory factor 5 (IRF5) in tumor progression: Prognostic and therapeutic potential. Biochim Biophys Acta Rev Cancer. (2024) 1879:189061. doi: 10.1016/j.bbcan.2023.189061. PMID: 38141865 PMC11977173

[83] Wang J Man K Ng KT . Emerging roles of C-C motif ligand 11 (CCL11) in cancers and liver diseases: Mechanisms and therapeutic implications. Int J Mol Sci. (2025) 26:4662. doi: 10.3390/ijms26104662. PMID: 40429807 PMC12111778

[84] Uchida T Nakagome K Hashimoto K Iemura H Shiko Y Mouri A . Eosinophils as predictive biomarkers in anti-programmed cell death 1 monotherapy for non-small cell lung cancer. Front Immunol. (2025) 16:1574314. doi: 10.3389/fimmu.2025.1574314. PMID: 41126830 PMC12537673

[85] Costantini S Dolzani P Panichi V Borzì RM Balaji P Daglia M . Inflaming and immune-resolving: The ambivalent role of eosinophils in osteoarthritis. Int J Mol Sci. (2025) 26:10948. doi: 10.3390/ijms262210948. PMID: 41303430 PMC12652785

[86] Poto R Marone G Ziegler SF Varricchi G . TSLP: Contrasting roles in cancer. Front Immunol. (2025) 16:1627235. doi: 10.3389/fimmu.2025.1627235. PMID: 40873585 PMC12378658

[87] Smolinska S Antolín-Amérigo D Popescu FD Jutel M . Thymic stromal lymphopoietin (TSLP), its isoforms and the interplay with the epithelium in allergy and asthma. Int J Mol Sci. (2023) 24:12725. doi: 10.3390/ijms241612725. PMID: 37628907 PMC10454039

[88] Sibille A Henket M Corhay JL Alfieri R Louis R Duysinx B . White blood cells in patients treated with programmed cell death-1 inhibitors for non-small cell lung cancer. Lung. (2021) 199:549–57. doi: 10.1007/s00408-021-00474-2. PMID: 34518898 PMC8510914

[89] Osawa H Shiozawa T Okauchi S Miyazaki K Kodama T Kagohashi K . Association between time to treatment failure and peripheral eosinophils in patients with non-small cell lung cancer treated with immune checkpoint inhibitors. Pol Arch Intern Med. (2021) 131:16049. doi: 10.20452/pamw.16049. PMID: 34180611

[90] Alves A Dias M Campainha S Barroso A . Peripheral blood eosinophilia may be a prognostic biomarker in non-small cell lung cancer patients treated with immunotherapy. J Thorac Dis. (2021) 13:2716–27. doi: 10.21037/jtd-20-3525. PMID: 34164164 PMC8182546

[91] Chu X Zhao J Zhou J Zhou F Jiang T Jiang S . Association of baseline peripheral-blood eosinophil count with immune checkpoint inhibitor-related pneumonitis and clinical outcomes in patients with non-small cell lung cancer receiving immune checkpoint inhibitors. Lung Cancer. (2020) 150:76–82. doi: 10.1016/j.lungcan.2020.08.015. PMID: 33080551

[92] Xu L Zheng Y Wang J Xu Y Xie Y Yang ZP . IL33 activates CD8+T and NK cells through MyD88 pathway to suppress the lung cancer cell growth in mice. Biotechnol Lett. (2020) 42:1113–21. doi: 10.1007/s10529-020-02815-2. PMID: 32140881

[93] Perales-Puchalt A Svoronos N Villarreal DO Zankharia U Reuschel E Wojtak K . IL-33 delays metastatic peritoneal cancer progression inducing an allergic microenvironment. Oncoimmunology. (2019) 8:e1515058. doi: 10.1080/2162402x.2018.1515058. PMID: 30546956 PMC6287802

[94] Cheng JN Luo W Sun C Jin Z Zeng X Alexander PB . Radiation-induced eosinophils improve cytotoxic T lymphocyte recruitment and response to immunotherapy. Sci Adv. (2021) 7:eabc7609. doi: 10.1126/sciadv.abc7609. PMID: 33514544 PMC7846170

[95] Blomberg OS Spagnuolo L Garner H Voorwerk L Isaeva OI van Dyk E . IL-5-producing CD4(+) T cells and eosinophils cooperate to enhance response to immune checkpoint blockade in breast cancer. Cancer Cell. (2023) 41:106–123.e110. doi: 10.1016/j.ccell.2022.11.014. PMID: 36525971

